# Supplementary material for: 6-month mortality and readmissions of hospitalized COVID-19 patients: A nationwide cohort study of 8,679 patients in Germany
Source: PLoS One. 2021 Aug 5;16(8):e0255427. doi: 10.1371/journal.pone.0255427 (PMC8341502; doi:10.1371/journal.pone.0255427)
Supplement: S1 Table — (DOCX) [file pone.0255427.s001.docx]

**S1 Table. Diagnosis codes of included patients**

| **Group** | | **Definition** |
| --- | --- | --- |
| Age group | |  |
| 18-59 years | | Age ≥ 18 & age ≤ 59 |
| 60-69 years | | Age ≥ 60 & age ≤ 69 |
| 70-79 years | | Age ≥ 70 & age ≤ 79 |
| 80 years and older | | Age ≥ 80 |
| Principal diagnosis at index hospitalization for inclusion | | ICD-10-GM  Principal diagnosis in: |
| Respiratory failure, pulmonary embolism, viral infection, sepsis or renal failure | | A41, B34, I26, J06, J09, J10, J11, J12, J15, J16, J17, J18, J20, J21, J22, J41, J44.0, J80, J96, J98, N17, R05, R06, U04 |
| Comorbidities & complications during index hospitalization | ICD-10-GM  Principal or secondary diagnosis in: | |
| ARDS | | J80.0 |
| BMI ≥ 40 | | E66.[]2 |
| Cognitive impairment | | F00, F01-05, F06.7, F06.9, F07.8-9, G30, G31.0-1, G31.9, G32, R41.0, U51 |
| COVID-19 | | U07.1! |
| Deep vein thrombosis | | I80.1-3, I82.2 |
| Delir, anoxia enzephalopathy, somnolence, sopor and coma | | F05, G93.1, G93.4, R40 |
| Intracerebral bleeding, cerebral infarction, stroke | | I61, I63, I64 |
| Lung edema | | J81 |
| Lung embolism | | I26 |
| Acute myocardial infarction | | I21, I24.8, I24.9 |
| Myocarditis | | I40, I41.1 |
| Renal failure | | N99.0, N17 |
| Septic shock | | A41, R65.0-1, R65.9, R57.2 |
| Readmissions | | ICD-10-GM  Principal or secondary diagnosis in: |
| Cardiovascular disorders/ complications | | I20, I21, I24.8, I24.9, I26, I33, I61, I63, I64, I80, I80.1-3, I82.2, I82.8, I82.9 |
| Gastrointestinal and liver disorders/ complications | | E87.2, K25.0-3, K26.0-3, K29.0-1, K72.0, K72.7, K72.9, K76.2, K76.3 |
| Neurological disorders/ complications | | F00-F05, F06.7, F06.9, F07.8-9, G30, G31.0-1, G31.9, G32, G93.1, G93.4, R40, R41.0, U51 |
| Renal disorders/ complications | | N17, N99.0 |
| Respiratory disorders/ complications | | J06, J09-18, J20-22, J41, J44.0, J80.0, J81, J95.2, J96.0, R05, R06, U04 |
| Systemic disorders/ complications | | A41, R57.2, R65.0-1, R65.9, D65, D68.8, D68.9, D69.5, D69.6 |
| Procedures during index admission | | Operationen- und Prozedurenschlüssel (OPS) in: |
| Dialysis | | 8-853, 8-854, 8-855, 8-857 |
| Tracheostomy | | 5-311, 5-312 |
| Extracorporeal membrane oxygenation | | 8-852.0 |
| Haemofiltration | | 8-853 |
| Invasive ventilation | | 8-701, 8-704, 5-311, 5-312 |
| Only non-invasive ventilation | | 8-706 & no invasive ventilation |
| Mechanical ventilation (non-invasive and invasive) | | 8-701, 8-704, 8-706, 5-311, 5-312 |
