# Supplementary material for: 6-month mortality and readmissions of hospitalized COVID-19 patients: A nationwide cohort study of 8,679 patients in Germany
Source: PLoS One. 2021 Aug 5;16(8):e0255427. doi: 10.1371/journal.pone.0255427 (PMC8341502; doi:10.1371/journal.pone.0255427)
Supplement: S2 Table — (DOCX) [file pone.0255427.s002.docx]

**S2 Table. Patient characteristics stratified by ventilation status**

| **Patients hospitalized for a COVID-19-related principal diagnosis** | **Total  (n = 8,679)** | **No ventilation**  **(n = 6,865)** | **Only non-invasive ventilation**  **(n = 206)** | **Invasive ventilation**  **(n = 1,608)** |
| --- | --- | --- | --- | --- |
|  |  |  |  |  |
|  |  | N (column percentage of total patients) | | |
| Male | 4,641 (53.5%) | 3,429 (49.9%) | 133 (64.6%) | 1,079 (67.1%) |
| Female | 4,038 (46.5%) | 3,436 (50.1%) | 73 (35.4%) | 529 (32.9%) |
| Age, years |  | (value refers to column total) | | |
| Mean (SD) | 68.6 (16.6) | 68.8 (17.4) | 70.3 (13.4) | 67.8 (12.8) |
| Median (IQR) | 72.0 (57.0 - 82.0) | 73.0 (57.0 - 83.0) | 72.5 (61.0 - 81.0) | 69.5 (59.0 - 78.0) |
| Age groups, years |  | N (column percentage of total patients) | | |
| 18-59 years | 2,451 (28.2%) | 2,005 (29.2%) | 43 (20.9%) | 403 (25.1%) |
| 60-69 years | 1,506 (17.4%) | 1,062 (15.5%) | 43 (20.9%) | 401 (24.9%) |
| 70-79 years | 1,905 (21.9%) | 1,357 (19.8%) | 53 (25.7%) | 495 (30.8%) |
| 80 years and older | 2,817 (32.5%) | 2,441 (35.6%) | 67 (32.5%) | 309 (19.2%) |
| Elixhauser comorbidities |  | N (column percentage of total patients) | | |
| Hypertension | 4,920 (56.7%) | 3,738 (54.5%) | 135 (65.5%) | 1,047 (65.1%) |
| Fluid and electrolyte disorders | 4,641 (53.5%) | 3,213 (46.8%) | 128 (62.1%) | 1,300 (80.8%) |
| Cardiac arrhythmias | 2,373 (27.3%) | 1,558 (22.7%) | 67 (32.5%) | 748 (46.5%) |
| Renal failure | 1,994 (23.0%) | 1,533 (22.3%) | 57 (27.7%) | 404 (25.1%) |
| Diabetes, uncomplicated | 1,912 (22.0%) | 1,335 (19.4%) | 52 (25.2%) | 525 (32.6%) |
| Congestive heart failure | 1,652 (19.0%) | 1,096 (16.0%) | 61 (29.6%) | 495 (30.8%) |
| Chronic pulmonary disease | 1,188 (13.7%) | 819 (11.9%) | 60 (29.1%) | 309 (19.2%) |
| Diabetes, complicated | 734 (8.5%) | 503 (7.3%) | 30 (14.6%) | 201 (12.5%) |
| Other neurological disorders | 671 (7.7%) | 487 (7.1%) | 15 (7.3%) | 169 (10.5%) |
| Coagulopathy | 623 (7.2%) | 197 (2.9%) | 18 (8.7%) | 408 (25.4%) |
| Depression | 601 (6.9%) | 441 (6.4%) | 17 (8.3%) | 143 (8.9%) |
| Liver disease | 411 (4.7%) | 148 (2.2%) | 7 (3.4%) | 256 (15.9%) |
| Pulmonary circulation disorders | 358 (4.1%) | 164 (2.4%) | 19 (9.2%) | 175 (10.9%) |
| Weight loss | 351 (4.0%) | 159 (2.3%) | 10 (4.9%) | 182 (11.3%) |
| Metastatic cancer | 53 (0.6%) | 44 (0.6%) | 1 (0.5%) | 8 (0.5%) |

Data are n (%), median (IQR) or mean (SD). BMI: Body Mass Index; ARDS: acute respiratory distress syndrome.

**Table S2 (continued). Patient characteristics stratified by ventilation status.**

| **Patients hospitalized for a COVID-19-related principal diagnosis** | **Total  (n = 8,679)** | **No ventilation**  **(n = 6,865)** | **Only non-invasive ventilation**  **(n = 206)** | **Invasive ventilation**  **(n = 1,608)** |
| --- | --- | --- | --- | --- |
|  |  |  |  |  |
| Further comorbidities |  | N (column percentage of total patients) | | |
| Cognitive impairment | 2,207 (25.4%) | 1,587 (23.1%) | 55 (26.7%) | 565 (35.1%) |
| Delir, anoxia encephalopathy, somnolence, sopor and coma | 1,058 (12.2%) | 533 (7.8%) | 37 (18.0%) | 488 (30.3%) |
| BMI ≥ 40 | 173 (2.0%) | 72 (1.0%) | 7 (3.4%) | 94 (5.8%) |
| Length of stay of index hospitalization |  | (value refers to column total) | | |
| Mean (SD) | 16.5 (19.5) | 11.9 (11.2) | 18.7 (15.5) | 35.7 (31.9) |
| Median (IQR) | 10.0 (6.0 - 20.0) | 9.0 (5.0 - 15.0) | 15.0 (8.0 - 26.0) | 27.0 (13.0 - 49.0) |
| Procedures during index hospitalization |  | N (column percentage of total patients) | | |
| Dialysis | 838 (9.7%) | 266 (3.9%) | 13 (6.3%) | 559 (34.8%) |
| Tracheostomy | 582 (6.7%) | 0 (0.0%) | 0 (0.0%) | 582 (36.2%) |
| Extracorporeal membrane oxygenation | 160 (1.8%) | 2 (0.0%) | 1 (0.5%) | 157 (9.8%) |
| Haemofiltration | 51 (0.6%) | 4 (0.1%) | 1 (0.5%) | 46 (2.9%) |
| Complications during index hospitalization |  | N (column percentage of total patients) | | |
| Septic shock | 1,406 (16.2%) | 438 (6.4%) | 41 (19.9%) | 927 (57.6%) |
| ARDS | 1,329 (15.3%) | 104 (1.5%) | 45 (21.8%) | 1,180 (73.4%) |
| Renal failure post procedure | 1,252 (14.4%) | 433 (6.3%) | 35 (17.0%) | 784 (48.8%) |
| Lung embolism | 188 (2.2%) | 75 (1.1%) | 5 (2.4%) | 108 (6.7%) |
| Intracerebral bleeding, cerebral infarction, stroke | 122 (1.4%) | 55 (0.8%) | 3 (1.5%) | 64 (4.0%) |
| Acute myocardial infarction | 112 (1.3%) | 44 (0.6%) | 5 (2.4%) | 63 (3.9%) |
| Deep vein thrombosis | 88 (1.0%) | 40 (0.6%) | 2 (1.0%) | 46 (2.9%) |
| Myocarditis | 38 (0.4%) | 16 (0.2%) | 1 (0.5%) | 21 (1.3%) |
| Lung edema | 15 (0.2%) | 4 (0.1%) | 1 (0.5%) | 10 (0.6%) |

Data are n (%), median (IQR) or mean (SD). BMI: Body Mass Index; ARDS: acute respiratory distress syndrome.
